# Periodontitis is associated with altered salivary anti-SARS-CoV-2 antibody patterns in vaccinated individuals

**DOI:** 10.3389/fimmu.2025.1737920

**Published:** 2026-01-14

**Authors:** Hassan Alkharaan

**Affiliations:** Department of Preventive Dental Sciences, College of Dentistry, Prince Sattam Bin Abdulaziz University, Al-Kharj, Saudi Arabia

**Keywords:** ELISA, periodontitis, salivary antibody, SARS-CoV-2, vaccines

## Abstract

**Background:**

Periodontitis is characterized by chronic inflammation and mucosal barrier disruption, which may influence salivary antibody dynamics after systemic vaccination. This study investigated the impact of periodontal disease severity on salivary anti-SARS-CoV-2 antibody responses in vaccinated, non-infected individuals.

**Methods:**

Saliva samples were collected from 258 SARS-CoV-2-naïve individuals who had received three vaccine doses. Participants were stratified into three groups based on a standardized oral examination: periodontally healthy controls (n=109), stage I/II periodontitis (n=93), and stage III/IV periodontitis (n=56). Anti-spike IgG, IgA, and sIgA levels were quantified using a validated in-house ELISA.

**Results:**

Salivary anti-spike IgG levels were significantly elevated in both stage I/II (*p* = 0.0002) and stage III/IV (*p* < 0.0001) periodontitis groups compared to controls, while IgA and sIgA levels did not differ between groups. IgG levels showed a strong positive correlation with periodontal bleeding scores (r=0.52, *p* < 0.0001). In contrast, sIgA did not correlate with bleeding (r=0.09, *p* = 0.12). Analysis of immunoglobulin isotype coordination revealed a strong positive correlation between IgG and IgA in controls (r=0.42, *p* < 0.0001) and stage I/II periodontitis (r=0.71, *p* < 0.0001), which was absent in stage III/IV periodontitis (r=0.25, *p* = 0.06).

**Conclusions:**

Periodontitis was associated with higher salivary anti-spike IgG binding levels, which also showed a strong positive correlation with periodontal bleeding. In contrast, IgA and sIgA binding levels did not differ across periodontal groups. The coordinated relationship between IgG and IgA observed in healthy individuals was disrupted in severe periodontitis. These findings demonstrate distinct alterations in salivary antibody patterns associated with periodontal disease severity following SARS-CoV-2 mRNA vaccination.

## 1 Introduction

Coronavirus SARS-CoV-2, a novel member of the *Coronaviridae* family, caused a global outbreak of COVID-19 in late 2019 and was subsequently declared a pandemic by the World Health Organization in March 2020 (1). Since then, the disease has infected over 770 million people worldwide and resulted in nearly 7 million deaths as of 2024 (2). Although the majority of cases are asymptomatic or mild, intensive care units were necessary for 5%, and hospitalization was observed in 14% of affected individuals (3, 4). Severe presentations are often associated with systemic complications, including acute respiratory distress syndrome, septic shock, and multiorgan failure (5). The primary driver of this deterioration is an excessive release of proinflammatory cytokines, commonly referred to as a “cytokine storm,” which contributes to widespread tissue damage, hyperinflammation, and immune dysregulation (6).

Multiple systemic comorbidities have been identified as major risk factors for severe COVID-19, including cardiovascular disease, diabetes mellitus, chronic kidney and lung disorders, liver disease, asthma, and obesity (3, 5). Poor oral health, particularly periodontitis, has been recognized as an additional contributor to poor outcomes (7–10). Periodontitis is a chronic, multifactorial inflammatory condition, affecting the supporting structures of the teeth and is strongly associated with systemic comorbidities such as diabetes, obesity, and cardiovascular disease (11–13). The overlap of risk factors between periodontitis and COVID-19 highlights the possibility of shared immunoinflammatory pathways that may influence both disease susceptibility and severity (7, 9).

At the molecular level, SARS-CoV-2 gains entry into host cells primarily through angiotensin-converting enzyme 2 (ACE2) and the transmembrane serine protease 2 (TMPRSS2), which are abundantly expressed in oral tissues, including the tongue epithelium, gingiva, periodontal pockets, salivary glands, and oral mucosa (14, 15). The widespread expression of these receptors underscores the potential role of the oral cavity as a site of viral uptake, replication, and transmission.

Emerging evidence further suggests a bidirectional relationship between periodontal disease and COVID-19 severity (7, 9, 16, 17). Individuals with periodontitis appear to be at increased risk of developing more severe outcomes, potentially through multiple biological mechanisms. Chronic periodontal inflammation contributes to sustained release of proinflammatory cytokines, which have been implicated in pulmonary tissue damage and heightened susceptibility to infection (18). Moreover, the dysregulated immune response associated with periodontitis may compromise host defense, thereby reducing the ability to effectively control SARS-CoV-2 infection (19). In addition, translocation of periodontal pathogens or their by-products into the lower respiratory tract may exacerbate pulmonary inflammation, further complicating the clinical course of COVID-19 (9, 19).

The mucosal immune system of the upper respiratory tract represents a critical first line of defense against SARS-CoV-2. Saliva has emerged as a valuable diagnostic fluid for monitoring mucosal antibody responses, even in mild or asymptomatic infections (15, 20, 21). Antibody responses generated by B cells at both systemic and mucosal sites have shown inverse associations with the risk of infection (21, 22). Vaccination against COVID-19 has been demonstrated to elicit robust increases in both serum and salivary SARS-CoV-2 specific IgG antibodies, which are sustained over extended periods (21–23). In contrast, mucosal IgA responses appear more transient, with stronger responses reported in individuals with prior SARS-CoV-2 exposure (21–23). Despite their short-lived nature, salivary IgA antibodies have been linked to enhanced protection against breakthrough infections, highlighting their importance in mucosal immunity (20–23).

In this study, we employed an in-house enzyme-linked immunosorbent assay (ELISA), previously validated for seroprevalence research (24, 25), to quantify spike-specific anti-SARS-CoV-2 IgG, IgA, and secretory IgA (sIgA) levels in saliva samples from COVID-19 non-infected, 3^rd^ dose vaccinated individuals with periodontal disease, and compared these responses with those of periodontally healthy controls. A comprehensive periodontal examination was performed for all participants, including assessment of gingival bleeding on probing (BOP, % of sites), probing depth (PD, mm), clinical attachment level (CAL, mm), and radiographic bone loss.

## 2 Materials and methods

### 2.1 Study design and sample collection

This study was approved by the Research Ethics Committee at Prince Sattam Bin Abdulaziz University (REC-HSD-114-2022) and conducted in accordance with the Declaration of Helsinki, with written informed consent obtained from all participants. Recruitment took place at the dental clinics of Prince Sattam Bin Abdulaziz University and King Saud Bin Abdulaziz University for Health Sciences (Riyadh, Saudi Arabia) between December 2021 and May 2022. A total of 258 saliva samples were collected from non-infected individuals who had received three doses of mRNA vaccines. Most participants (93.5%) received three doses of Pfizer-BioNTech (BNT162b2), whereas 6.5% received a mixed schedule consisting of two Pfizer-BioNTech doses followed by a Moderna (mRNA-1273) booster. Participants were stratified into three cross-sectional groups based on periodontal status (26): Periodontal health controls (n = 109), periodontitis stage I–II (n = 93), and stage III–IV (n = 56). Periodontal assessments were performed by three blinded and calibrated examiners, with calibration undertaken by re-examining a randomly selected quadrant in ten patients, and inter-examiner reliability assessed using coefficients of variation (CVs) and intraclass correlation coefficients (ICCs) based on a two-way random-effects model with absolute agreement. Demographic and clinical data were collected from hospital records and standardized questionnaires (**Supplementary File**), while COVID-19 infection and vaccination history were verified through the Ministry of Health mobile application (Tawakkalna), with infection confirmed by SARS-CoV-2 RT-PCR. Participants with gingivitis, immunosuppressive conditions, older than 60 years, hospitalization due to COVID-19, or autoimmune disorders were excluded.

Unstimulated saliva was collected under professional supervision before dental treatment. Participants refrained from eating, drinking, smoking, or oral hygiene for at least one hour. Saliva was passively drooled into sterile 2 mL tubes and stored at −80°C within 12 hours.

### 2.2 SARS-CoV-2 antibody detection by ELISA

An in-house ELISA was performed to detect salivary IgG, IgA, and secretory IgA (sIgA) against SARS-CoV-2, adapted from a previously described protocol (25) with minor modifications. Briefly, Nunc MaxiSorp 96-well plates (Thermo Fisher, Waltham, MA, USA) were coated with recombinant SARS-CoV-2 spike S1 subunit (Sino Biological, Beijing, China; Cat. No. 40591-v08B1) at 1 μg/mL and incubated overnight at room temperature (RT). Plates were then washed six times with phosphate-buffered saline containing 0.05% Tween-20 (PBS-T) using an automated washer (Molecular Devices, San Jose, CA, USA) and blocked with 100 μL PBS-T containing 10% skimmed milk for 1 h at RT. Saliva samples were diluted 1:30 in PBS-T, and 50 μL of each sample was added in duplicate wells and incubated for 2 h at RT. For IgG and IgA detection, 50 μL of alkaline phosphatase–conjugated goat anti-human IgG or IgA antibody (Thermo Fisher, Waltham, MA, USA; 1:1000) was applied for 1 h at RT. For sIgA detection, wells were incubated with mouse anti-human secretory component antibody (Calbiochem HP6141, 1 µg/mL) for 1 h, followed by alkaline phosphatase–conjugated goat anti-mouse IgG (1:1000) for 1 h at RT. Plates were washed before adding p-nitrophenylphosphate (PNPP) substrate in diethanolamine buffer, and absorbance was measured at 405 nm using a microplate reader (Molecular Devices, San Jose, CA, USA). Cut-off values were defined as the mean optical density (OD) of six pre-pandemic negative control samples plus six standard deviations (SD), a highly stringent threshold selected to ensure maximal specificity, consistent with our previously validated evaluation approach (21). Negative controls were saliva collected prior to the COVID-19 pandemic, and positive controls were saliva from convalescent COVID-19 cases. The OD measurements of internal controls were used to calculate the inter/intra-assay coefficients of variation (CV) for all runs. The intra-assay CV was 4.6 ± 2.5%, and the inter-assay CV was 7.5 ± 3.6% Aliquoted control samples were included in each assay run to minimize freeze–thaw variability.

### 2.3 Commercial kit validation

The in-house assay was validated using a commercial anti-SARS-CoV-2 IgG ELISA kit (Euroimmun, Schleswig-Holstein, Germany) according to the manufacturer’s instructions. Optical density (OD) values were expressed as sample-to-calibrator ratios, and positive and negative controls supplied with the kit were included in each run. Validation was performed using saliva samples spanning the full range of antibody values and representing all periodontal study groups, with each sample measured in duplicate.

### 2.4 Statistical analyses

Statistical analyses were performed using GraphPad Prism version 9.0. Pairwise group comparisons were conducted using the Kruskal–Wallis test with Dunn’s multiple comparisons correction for quantitative data and Fisher’s exact test for qualitative data. Associations between anti-spike IgG, IgA, levels and bleeding scores were evaluated using Spearman’s rank correlation. Inter-examiner reliability and the inter/intra-ELISA assay was assessed using coefficients of variation (CVs).

## 3 Results

### 3.1 Characteristics of the study participants

A total of 258 saliva samples were collected from vaccinated individuals and classified into three groups according to periodontal status: healthy controls (n = 109), stage I–II periodontitis (n = 93), and stage III–IV periodontitis (n = 56). Participants in the stage III–IV group were older compared to those in the stage I–II group, whereas no significant differences were observed across the groups for other demographic or clinical parameters (**Table 1**).

**Table 1:** Demographics of study participants.

Parameters	Control (*n* = 109)	Periodontitis stage I or II (*n* = 93)	Periodontitis stage III or IV (*n* = 56)
Gender (F:M)	39:70	31:62	19:37
Age (years) median (range)	36 (19-57)	37 (18-59) ^*Ω^	40 (27-56) ^**Φ^
BMI (kg/m^2^) median (range)	24.5 (18.3-43.2)	26.4 (15.6-42)	24.8 (16.8-44.5)
Smoking (%)	18.4	20.4	25
Antibiotic (%) (<3month)	5.5	6.4	12.5
Diabetes (%)	7.4	16.1	17.9
Days since 3rd dose median (range) §	84 (31-114)	83 (26-112)	80 (36-115)

Pairwise statistical comparisons between each group were made using Kruskal-Wallis test with Dunn’s multiple comparisons correction for quantitative parameters and Fisher’s exact test for qualitative values. Bold indicates statistical significance (* *P* <.05, ** *P* <.01). § indicates time interval between sample collection and last dose. Ω Indicates comparison with Periodontitis stage III & IV group. Φ indicates comparison with control group.

### 3.2 The examination and assay validation

**Figure 1:**
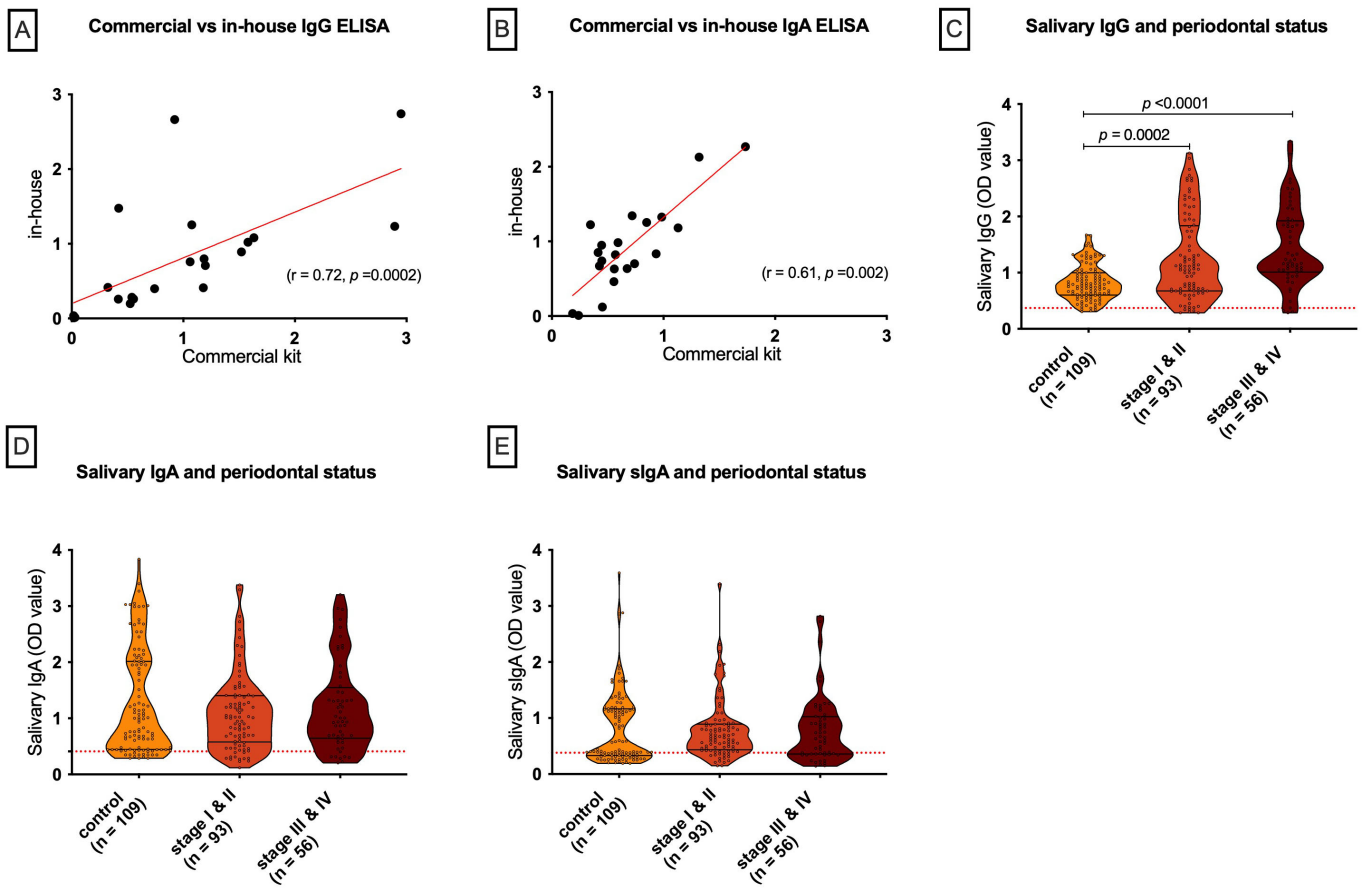
Correlation of in-house ELISA and salivary antibody levels by periodontal status. **(A, B)** In-house ELISA results strongly correlated with commercial kits for salivary anti-spike IgG and IgA. **(C–E)** Salivary anti-spike IgG was significantly higher in stage I–II and stage III–IV periodontitis compared with controls **(C)**, whereas IgA **(D)** and sIgA **(E)** showed no group differences. Violin plots display individual values; dashed lines indicate assay cut-offs.

Inter-examiner reliability testing demonstrated a high level of consistency among the three calibrated examiners. For probing depth, the coefficient of variation was 9%, while for clinical attachment loss, the coefficient of variation was 11% with an ICC of 0.89, indicating excellent agreement across examiners. The in-house ELISA was further validated against a commercial kit using 21 saliva samples including the negative samples, showing strong positive correlations for both IgG (r = 0.72, 95% CI: 0.41–0.88, *p* = 0.0002) and IgA (r = 0.61, 95% CI: 0.28–0.85, *p* = 0.002) (**Figure 1B**), confirming the assay’s reliability.

### 3.3 Salivary antibodies and periodontitis severity

Salivary IgG, but not IgA or sIgA, was associated with periodontitis severity. IgG levels were significantly higher in stage I/II (*p* = 0.0002) and stage III/IV (*p* < 0.0001) compared with controls, with no difference between stage I/II and stage III/IV (*p* = 0.07) (**Figure 1C**). In contrast, IgA and sIgA levels showed no significant differences across groups (all *p* > 0.35) (**Figures 1D, 1E**, respectively).

### 3.4 Correlation of salivary antibodies with periodontal bleeding scores

**Figure 2:**
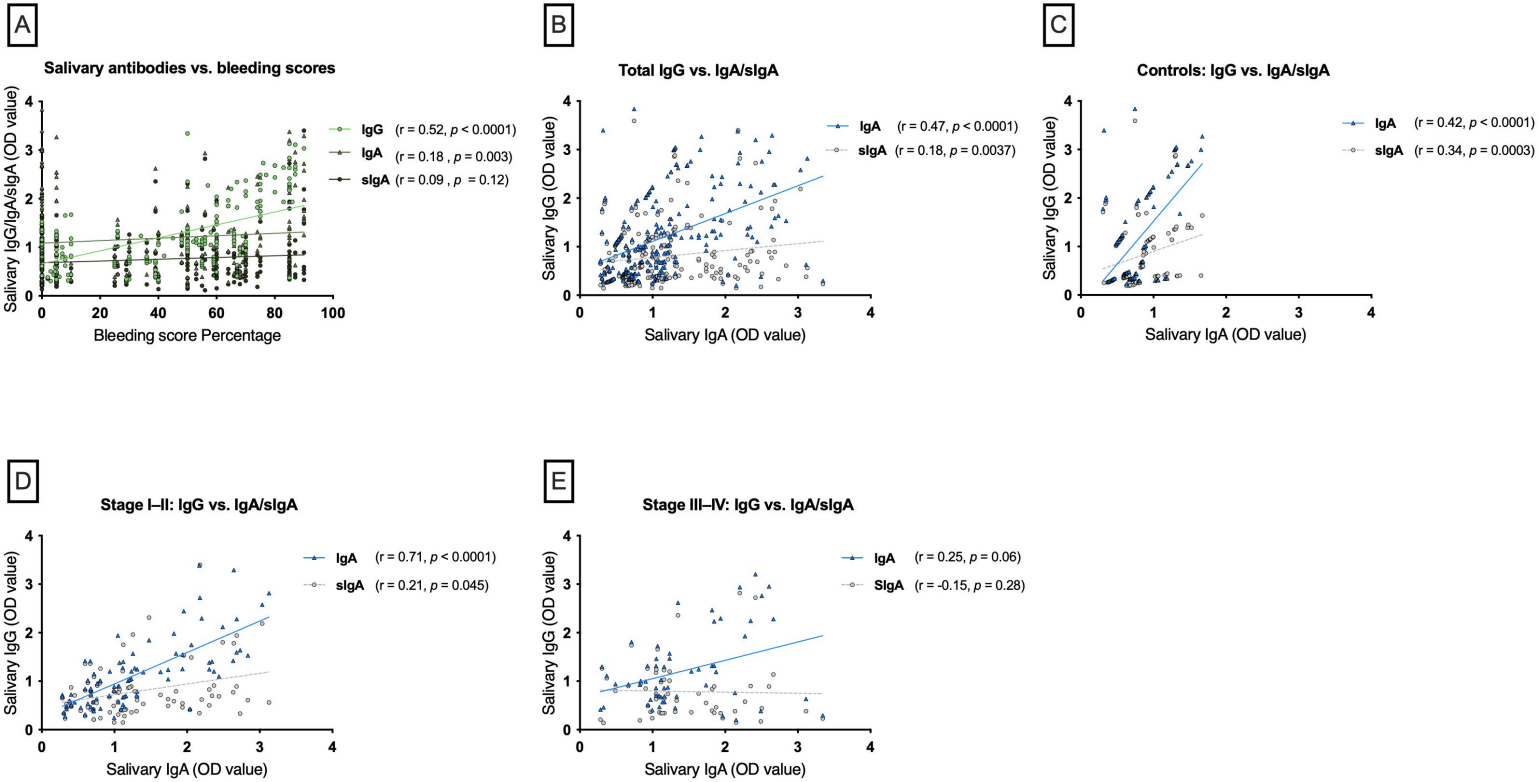
Correlations of salivary antibodies with bleeding and between isotypes by periodontal status. **(A)** Salivary anti-spike IgG correlated strongly with bleeding scores, while IgA and sIgA showed weaker or no correlations. **(B)** Overall, IgG correlated positively with IgA and weakly with sIgA. **(C)** In controls, IgG correlated with both IgA and sIgA. **(D)** In stage I–II periodontitis, IgG showed a strong correlation with IgA and a weak correlation with sIgA. **(E)** In stage III–IV periodontitis, these correlations were lost.

To assess whether elevated IgG levels in periodontitis may result from translocation through periodontal bleeding, Spearman correlations between salivary antibody levels and bleeding scores were examined. Salivary anti-spike IgG showed a strong positive correlation with bleeding scores (r = 0.52, *p* < 0.0001). IgA also correlated positively, though more weakly (r = 0.18, *p* = 0.003). In contrast, sIgA levels were not significantly associated with bleeding scores (r = 0.09, *p* = 0.12) (**Figure 2A**).

### 3.5 Correlation of salivary antibodies with periodontal status

Spearman correlation analysis was performed to evaluate the relationship between different salivary antibody classes in the study cohort and its subgroups based on periodontal status. In the total cohort, salivary anti-spike IgG levels showed a strong positive correlation with IgA (r = 0.47, *p* < 0.0001) and a weaker but significant correlation with sIgA (r = 0.18, *p* = 0.0037) (**Figure 2B**). A similar trend was observed in the control group, where salivary IgG positively correlated with IgA (r = 0.42, *p* < 0.0001) and sIgA (r = 0.34, *p* = 0.0003) (**Figure 2C**). Further analysis of the periodontitis subgroups revealed distinct patterns. In patients with mild-to-moderate periodontitis (Stages I&II), a very strong positive correlation was found between salivary IgG and IgA levels (r = 0.71, *p* < 0.0001) and a significant but weak correlation was also observed between IgG and sIgA (r = 0.21, *p* = 0.045) (**Figure 2D**). In contrast, no statistically significant correlation was found between salivary IgG and either IgA (r = 0.25, *p* = 0.06) or sIgA (r = −0.15, *p* = 0.28) in the severe periodontitis group (Stages III&IV) (**Figure 2E**).

### 3.6 Multivariable analysis of demographic and clinical correlates of salivary antibody levels

To assess whether the observed associations between periodontal status and salivary antibody patterns were influenced by demographic or clinical covariates, multivariable linear regression analyses were conducted (**Supplementary Table S1**). Models were adjusted for age, sex, body mass index, smoking status, antibiotic use, diabetes, and time elapsed since the third vaccine dose. After adjustment, none of the examined covariates showed a statistically significant association with salivary anti-spike IgG or sIgA binding levels. In contrast, smoking status remained independently associated with higher salivary anti-spike IgA levels (β = 0.349, 95% CI 0.094–0.604; p = 0.008).

## 4 Discussion

This study investigated the relationship between periodontal disease severity and salivary antibody responses following 3^rd^ dose of mRNA SARS-CoV-2 vaccination in non-infected COVID-19 individuals. Three principal findings emerged: first, salivary anti-spike IgG levels, but not IgA or sIgA, were significantly elevated in individuals with periodontitis compared with periodontally healthy controls. Second, salivary IgG levels showed a strong positive correlation with periodontal bleeding scores. Third, the coordinated correlation between IgG and IgA observed in healthy and mild-moderate (Stage I/II) periodontitis groups was absent in individuals with severe (Stage III/IV) periodontitis. Collectively, these findings indicate that periodontitis, particularly in its advanced stages, is associated with altered salivary anti–SARS-CoV-2 antibody patterns in vaccinated individuals.

The most intriguing finding of this study is the significant elevation of salivary anti-spike IgG in individuals with periodontitis. IgG in saliva is generally regarded as being largely derived from the systemic circulation (27), and the strong positive correlation between salivary IgG levels and periodontal bleeding score (r = 0.52, p < 0.0001) in our cohort supports the possibility that inflamed and ulcerated sulcular epithelium may facilitates increased passage of serum-derived IgG into the oral cavity (27, 28). Nevertheless, serum anti-spike IgG titers, gingival crevicular fluid (GCF) antibodies, and epithelial permeability markers such as albumin or lactoferrin, which have been used to assess transudation across inflamed periodontal tissues (28), were not measured. Consequently, the extent to which elevated salivary IgG reflects passive serum leakage versus other systemic or local factors cannot be determined. The serum-leakage mechanism should therefore be viewed as a plausible working hypothesis rather than a definitive explanation, and future studies incorporating paired serum-saliva-GCF sampling and barrier integrity markers will be required to test this directly.

In contrast to IgG, the levels of salivary IgA and sIgA did not differ significantly across periodontal health groups. sIgA, the predominant dimeric IgA form in oral secretions, is actively produced by mucosal plasma cells and transported to epithelial surfaces, where it constitutes a principal immunological barrier at mucosal surfaces (29). Prior studies have demonstrated the superior neutralizing capacity of dimeric IgA compared with monomeric IgA and IgG against SARS-CoV-2 (20, 30), underscoring the relevance of mucosal sIgA responses in vaccine-induced immunity. The stability of IgA and sIgA binding levels observed in our cohort can therefore be framed as quantitatively preserved mucosal IgA output despite periodontal inflammation. Although transforming growth factor-β (TGF-β) and interleukin-6 (IL-6), both of which are upregulated in periodontal inflammation, are central regulators of IgA class-switch recombination and plasma-cell differentiation (31, 32), enhanced cytokine signaling does not necessarily translate into increased salivary IgA concentrations, as effective mucosal IgA secretion depends on additional processes including plasma-cell homing, J-chain–dependent polymerization, and polymeric immunoglobulin receptor–mediated epithelial transport (27, 31). Chronic inflammatory and dysbiotic environments, such as those characteristic of periodontitis, may perturb these tightly regulated steps, resulting in preserved bulk IgA levels while altering coordination or functional quality. At the same time, our findings speak only to IgA quantity, not functional quality, as we did not assess neutralizing activity, nor did we measure cytokine milieu or plasma-cell homing pathways. Consistent with the compartmental regulation, IgA showed only a weak association with bleeding (r = 0.18, p = 0.003), and sIgA showed no significant association (r = 0.09, p = 0.12), in contrast to IgG, which is more dependent on vascular permeability (27, 28).

Perhaps the most novel aspect of this study is the disrupted antibody isotype coordination observed in severe periodontitis. In healthy individuals and those with mild–moderate periodontitis (stages I/II), salivary IgG and IgA levels were strongly correlated, indicating coordinated salivary antibody patterns across isotypes potentially mediated by shared regulatory networks or aligned plasma-cell differentiation and homing pathways. In severe periodontitis (Stages III/IV), however, this relationship was no longer evident: correlations between IgG and IgA became non-significant, and the IgG–sIgA association trended inversely. Such a disruption aligns with the profound immunopathology of advanced periodontitis, which is characterized by a dysbiotic oral microbiome and sustained hyper-inflammatory responses (31). Multi-omics studies further demonstrate that periodontal dysbiosis alters not only microbial composition but also the metabolic and immunomodulatory capacity of the oral microbiome, thereby reshaping local immune signaling networks (33, 34). Within such inflammatory environments, cytokines such as TNF-α, IL-1β, and IL-6, known to be elevated in severe periodontitis, may influence plasma-cell activity or affect epithelial transport pathways relevant to IgA secretion (31, 32). In addition, mucosal immunology literature indicates that effective secretory IgA responses depend on coordinated plasma-cell localization, J-chain–dependent assembly, and polymeric immunoglobulin receptor (pIgR)–mediated epithelial transport; perturbations in these tightly regulated processes within chronically inflamed tissues could contribute to altered antibody coordination (32). Taken together, these microbial and immunological considerations provide a biologically plausible framework for interpreting the loss of IgG–IgA coordination in severe periodontitis as reflecting altered salivary antibody organization, rather than a simple quantitative variation or direct evidence of systemic immune dysregulation.

These findings parallel evidence from the gut, where microbiota composition strongly influences systemic vaccine responses. Animal models have shown that germ-free or antibiotic-treated mice exhibit reduced antibody production and impaired T-cell activation following influenza vaccination (35, 36). Such data emphasize the critical role of host–microbiota interactions in shaping both mucosal and systemic immunity. By extension, dysbiotic oral microbial communities may exert underappreciated effects on vaccine responsiveness, immune coordination, and antibody dynamics (37, 38). In this context, the IgG–IgA decoupling observed in severe periodontitis (stages III/IV) may represent a candidate biomarker of mucosal immune dysregulation, offering a novel perspective on how chronic oral inflammation could influence vaccine-related immune pathways.

This study has several notable strengths, including a relatively large sample size, stratification by well-defined periodontal stages, and rigorous periodontal examination (high ICCs). The use of validated in-house ELISAs with high concordance to a commercial kit further strengthens the analytical robustness of the findings. Several methodological decisions were also intentionally undertaken to mitigate potential confounders—for example, restricting inclusion to triple-vaccinated individuals minimized variability in vaccine exposure, and excluding gingivitis cases provided clearer contrast between periodontal health and established periodontitis. Nonetheless, several limitations should be acknowledged. First, the cross-sectional design precludes causal inference. Elevated salivary IgG levels may reflect passive transudation from inflamed periodontal tissues, higher systemic antibody titers, or other unmeasured host factors; paired serum, gingival crevicular fluid (GCF), and epithelial permeability markers were not collected, although the strong correlation between salivary IgG and bleeding scores provides supportive contextual evidence. Second, antibody binding levels were quantified without assessment of neutralizing activity or IgA subclass distribution, limiting conclusions regarding the functional quality of mucosal immunity. Third, the assay targeted the spike protein of the ancestral Wuhan-Hu-1 strain; however, despite ongoing viral evolution under immune pressure (39), vaccine-induced antibodies have been shown to recognize conserved epitopes and exhibit cross-reactivity across emerging variants (40–42). Although age differed significantly between periodontal groups, consistent with the epidemiology of periodontitis, multivariable analyses adjusting for age and other demographic and clinical variables did not identify age as an independent correlate of salivary anti-spike antibody levels, suggesting that age was unlikely to account for the observed antibody patterns. In addition, saliva flow rate and total protein normalization were not performed due to sample-volume constraints, although standardized collection protocols were applied to minimize variability. Finally, we did not assess local factors such as oral microbiota, proteases, or mucosal cytokines, all of which are increasingly recognized as critical modulators of salivary antibody stability and production (43).

Future longitudinal studies should examine antibody dynamics before and after periodontal therapy to clarify causal relationships. Mechanistic investigations into the homing of vaccine-induced plasma cells to oral tissues and the influence of periodontal inflammation on mucosal immune coordination are also warranted.

## 5 Conclusions

In vaccinated, non-infected individuals, salivary anti-spike IgG binding levels, but not IgA or sIgA, were associated with periodontitis and correlated with bleeding scores, reflecting the close link between periodontal inflammation and salivary antibody patterns. The distinct isotype correlation profiles observed across periodontal disease stages suggest that advancing periodontal inflammation may influence systemic–mucosal vaccine-induced antibody relationships; however, determining any functional consequences for mucosal IgA will require additional assessments such as neutralization assays. Collectively, these findings emphasize the importance of accounting for periodontal health status when interpreting salivary antibody measurements in vaccinated individuals and highlight the need for future studies that integrate serum, saliva, and GCF antibody profiles with key mucosal biological factors including the oral microbiota, protease activity, and cytokine signaling to more fully contextualize salivary antibody responses within oral and systemic immunity.

## Data Availability

The raw data supporting the conclusions of this article will be made available by the authors, without undue reservation.
