# Supplementary material for: Periodontitis is associated with altered salivary anti-SARS-CoV-2 antibody patterns in vaccinated individuals
Source: Front Immunol. 2026 Jan 14;16:1737920. doi: 10.3389/fimmu.2025.1737920 (PMC12847319; doi:10.3389/fimmu.2025.1737920)
Supplement: Supplementary file 1 [file Table1.docx]

**Supplementary Table S1.** Multivariable Linear Regression Analysis of Salivary Anti-Spike Antibody Levels (IgG, IgA, and sIgA)

| Variable | IgG β  (95% CI) | *p* Value | IgA β  (95% CI) | *p* Value | sIgA β  (95% CI) | *p* Value |
| --- | --- | --- | --- | --- | --- | --- |
| Gender (male) | 0.012  (−0.158–0.182) | 0.888 | −0.004  (−0.218–0.211) | 0.973 | 0.047  (−0.102–0.196) | 0.534 |
| Age (years) | 0.007  (−0.002–0.015) | 0.138 | 0.001  (−0.009–0.012) | 0.801 | −0.002  (−0.010–0.005) | 0.580 |
| BMI (kg/m²) | 0.002  (−0.011–0.014) | 0.816 | 0.009  (−0.007–0.025) | 0.285 | 0.006  (−0.005–0.017) | 0.288 |
| Smoking | 0.020  (−0.182–0.222) | 0.848 | **0.349**  **(0.094–0.604)** | **0.008** | 0.167  (−0.011–0.344) | 0.066 |
| Antibiotic use (<3month) | −0.254  (−0.563–0.056) | 0.107 | −0.115  (−0.505–0.276) | 0.564 | 0.021  (−0.251–0.293) | 0.880 |
| Diabetes | 0.062  (−0.180–0.304) | 0.612 | −0.150  (−0.456–0.156) | 0.335 | −0.033  (−0.245–0.180) | 0.763 |
| Days since 3rd dose | 0.001  (−0.003–0.004) | 0.772 | 0.001  (−0.003–0.006) | 0.562 | 0.001  (−0.002–0.005) | 0.369 |

Multivariable linear regression models assessing demographic and clinical predictors of salivary anti-spike IgG, IgA, and secretory IgA (sIgA) binding levels. **β** represents the regression coefficient indicating the estimated change in antibody binding level per unit change in each independent variable. CI denotes the 95% confidence interval. All models were adjusted for the variables listed. Bold font indicates statistical significance (p < 0.05).
